# Pregabalin Treatment does not Affect Amyloid Pathology in 5XFAD Mice

**DOI:** 10.2174/1567205018666210713125333

**Published:** 2021

**Authors:** Katherine R. Sadleir, Jelena Popovoic, Wei Zhu, Cory T. Reidel, Ha Do, Richard B. Silverman, Robert Vassar

**Affiliations:** 1 Department of Neurology, Feinberg School of Medicine, Northwestern University, Chicago, IL 60611, USA; 2 Department of Chemistry, Chemistry of Life Processes Institute, Center for Molecular Innovation and Drug Discovery, Center for Developmental Therapeutics, Northwestern University, Evanston, IL 60611, USA; 3 Department of Pharmacology, Feinberg School of Medicine, Northwestern University, Chicago, IL 60611, USA

**Keywords:** Alzheimer’s, amyoid, pregabalin, synthesis, BACE1, dystrophic neurites, calcium, 5XFAD

## Abstract

**Background:**

Calcium dysregulation has been proposed to play a causative role in the development of Alzheimer’s disease pathology. Pregabalin is a compound already approved for human use, marketed as the prescription drug Lyrica. It binds the α2-δ subunit of P/Q-type voltage-gated calcium channels, lowering calcium influx and providing effective treatment for epilepsy and neuropathic pain.

**Objective:**

We hypothesize that increased resting calcium in neuronal processes near amyloid plaques plays a role in the development of neuritic dystrophies and further progression of amyloid pathology.

**Methods:**

5XFAD mice were treated orally for 12 weeks with pregabalin, then immunoblotting and immunofluorescent imaging were used to quantify neuritic dystrophy and amyloid deposition in pregabalin compared to placebo-treated mice.

**Results:**

The treatment did not decrease markers of neuritic dystrophy or amyloid deposition. The image analysis of neuritic dystrophy on a plaque-by-plaque basis showed a small non-significant increase in the relative proportion of LAMP1 to Aβ42 in plaques with areas of 50-450 μm^2^ in the cortex of pregabalin-treated mice. In addition, there was a statistically significant positive correlation between the measured cerebral concentration of pregabalin and the relative levels of BACE1 and Aβ in the cortex. This relationship was not observed in the hippocampus, and there was no increase in average Aβ levels in pregabalin treated mice compared to placebo. We confirmed previous findings that smaller amyloid plaques are associated with a greater degree of neuritic dystrophy.

**Conclusion:**

Pregabalin may have an effect on Aβ that merits further investigation, but our study does not suggest that pregabalin contributes substantially to amyloid pathology.

## 1. INTRODUCTION

Alzheimer’s disease (AD) affects a high proportion of the aging population, and despite extensive research, the causes of the disease are not fully understood, and no disease-modifying treatment is available. Alzheimer’s disease is characterized by two hallmark pathologies, *i.e.*, amyloid plaques and tau tangles [1, 2]. Plaque-forming amyloid-β (Aβ) is generated by the sequential cleavage of Amyloid-Precursor Protein (APP) by first the β-secretase, β-APP Cleaving Enzyme (BACE1) [3-6], and then presenilin, the active subunit of the γ-secretase enzyme complex. The amyloid cascade hypothesis [7], which states that amyloid deposition precedes the neurotoxic tau tangles and may promote their formation, leading to synaptic dysfunction and memory loss, is well supported by genetic, cell biological, and animal model data, the many therapeutics that have targeted the secretase enzymes and Aβ themesleves, have so far not proven successful in clinical trials.

Another long-standing hypothesis of AD etiology, complementary to the amyloid hypothesis, is the calcium hypothesis, first advanced in 1976 [8], which states that the pathologies and risk factors associated with AD, through various mechanisms, cause dysregulation of [Ca^+2^]_i_ in neurons, and this leads to a degradation of the functionality of the neuron through effects, such as spine loss, dysfunction of kinases and phosphatases, synaptic dysfunction, and eventually neuron loss. The current calcium hypothesis, as outlined in 2017 [9], posits that “dysregulation of Ca^2+^ is the final common event, leading to the decline in the normal functions of a neuron, and eventually its demise”. This makes the maintenance of calcium homeostasis an appealing therapeutic approach that could benefit familial early-onset AD caused by genetic mutations and late-onset AD associated with both genetic and environmental factors. Despite the compelling nature of the calcium hypothesis, there is a surprising lack of consensus regarding the mechanism of calcium dyshomeostasis in AD or whether dysregulation of calcium definitively contributes to the pathophysiology observed in AD.

One possible mechanism of calcium dysregulation is via toxicity of Aβ in the form of both soluble oligomers and insoluble plaques. There are many mechanisms by which Aβ has been hypothesized to cause increased intracellular calcium, including activating NMDA receptors [10], creating pores in the membrane [11], and interaction with calcium channels [12], *etc.* Previous reports indicate that in a mouse model of amyloid pathology, free calcium levels are elevated in neurons in regions immediately surrounding plaques but are normal in regions distant from plaques and in pre-plaque mice [13]. Altered neuronal firing patterns have been reported in the hippocampus and cortex around plaques, with many neurons in the immediate vicinity of plaques being hyperactive [14, 15], suggesting altered calcium homeostasis in these regions.

We have hypothesized that calcium dysregulation in the processes around plaques causes peri-plaque damage to microtubule networks and disrupts vesicle trafficking [16]. These dystrophic neurites are detrimental in two ways. First, they are sites of increased Aβ generation. BACE1 is elevated in the brains of A,D patients [17-21] and mouse models of amyloidosis, specifically in presynaptic dystrophic axons and terminals around amyloid plaques [21, 22]. Additionally, APP and PS1 [23] are found in these dystrophic neurites, along with BACE1-cleaved APP fragments, sAPPβ, Aβ42, and Aβ_40_ [16]. These localities of increased amyloid generation lead to increased plaque seeding in the immediate vicinity of existing plaques [24]. Additionally, although these dystrophic axons contain some presynaptic markers, they are missing others [16] and are unlikely to contribute to synaptic plasticity [25]. Since a high degree of BACE1 inhibition appears to have negative side effects [26-28], it is an appealing potential therapeutic strategy to prevent BACE1 elevation specifically in the vicinity of the plaques, thus avoiding side effects and helping the blockage or clearance of dystrophies. Several lines of evidence indicate that neuritic dystrophies are dynamic and changing structures still they are connected to living neuronal cell bodies [29]. Several groups report growth, shrinkage, and other changes in dystrophic neurites [30, 31], and even their disappearance, as plaques, are cleared by treatment with anti-amyloid antibody [32].

One potential approach to prevent or remove dystrophic neurites is to reduce intraneuronal resting calcium using currently available drugs that target various calcium channels in the brain. In this report, we test the capacity of the FDA-approved drug pregabalin to reduce BACE1 levels, dystrophic neurites, and amyloid plaques in the 5XFAD mouse model of AD. Pregabalin, first synthesized in 1991 [33], and marketed by Pfizer as the drug Lyrica for epilepsy and neuropathic pain, binds specifically to the α2-δ1 and α2-δ2 auxiliary subunits of P/Q-type voltage-gated calcium channels [34, 35]. The α2-δ1 subunit is widely expressed in the brain, while α2-δ2 is mainly found in GABAergic neurons, especially cerebellum [36]. The α2-δ1 auxiliary subunit is localized to presynaptic regions, such as the mossy fiber terminals of CA3 [37] and dendrites and cell bodies [38, 39]. The α2-δ1 and α2-δ2 subunits play a role in trafficking the pore forming α-subunits of the calcium channel to the membrane and modulate neuronal conductivity [40]. Several reports indicate that pregabalin lowers calcium influx in synaptosome preparations, and in electrophysiological studies, pregabalin decreases the release of various neurotransmitters [41]. These properties help explain pregabalin’s anticonvulsant effects, as well as its efficacy in the treatment of neuropathic pain and anxiety. Here, we tested the hypothesis that pregabalin is able to ameliorate dystrophic neurites and amyloid pathology, potential consequences of calcium dysregulation in AD, in the 5XFAD mouse model.

## 2. MATERIALS AND METHODS

### 2.1. Pregabalin Synthesis

#### 2.1.1. Chemistry

**Scheme 1.**
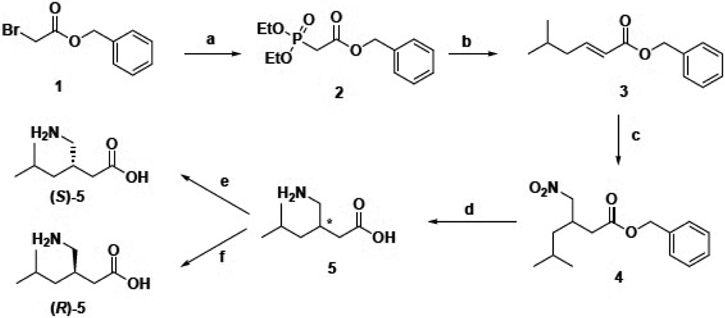
Synthetic route for pregabalin. Reagents and Conditions: (**a**) P(OEt)_3_, neat, 150 °C, 3 h; (**b**) K_2_CO_3_, isovaleradehyde, H_2_O, 40 °C, 2 d; (**c**) CH_3_NO_2_, TMG, 2 d; (**d**) PdOH (10%), MeOH, H_2_ balloon, rt, overnight; (**e**) (*S*)-mandelic acid, *^i^*PrOH (3% water), THF/H_2_O; (**f**) (*R*)-mandelic acid, *^i^*PrOH (3% water), THF/H_2_O; For complete description, supplemental methods need to be reviewed.

The synthetic route for pregabalin is shown in Scheme 1. Benzyl bromoacetate (**1**) was heated with triethyl phosphite to give **2** in quantitative yields, which subsequently underwent a Wadsworth-Emmons reaction with isovaleraldehyde to give **3**. The treatment of **3** with nitromethane and tetramethylguanidine yielded **4**, which was deprotected and reduced by Pd-catalyzed hydrogenation. The enantiomers were afforded by the chiral resolution [42] of racemic pregabalin (**5**) with *(S)*- and *(R)*-mandelic acids.

#### 2.1.2. Mice

5XFAD mice were generated and genotyped as described in a study [43]. 5XFAD positive males were bred to B6SJL F1 hybrid females to generate one large cohort of 5XFAD and non-transgenic littermates of the same age used in this experiment. After the mice were 9 weeks old, they were provided with water bottles containing 0.25 mg/ml pregabalin or placebo. The chosen placebo was the *R*-enantiomer of pregabalin that is ten times less potent in binding the α2-δ subunit of the P/Q calcium channel receptor, and has no reported physiological effects [44]. In our study, the average mouse weighed between 21 and 28 grams, depending on age and sex. Water consumption of pregabalin-treated mice averaged 5.5 ml/day, so the daily dose was varied roughly from 35-60 mg/kg/day. This was close to our target of 50 mg/kg/-day, which was the NOAEL (no observed adverse effect level) in rats for pregabalin [45]. Pregabalin or placebo solution was refilled with the solution that was prepared fresh each week. The number of animal that were used as follows: Placebo non-transgenic: 7 female and 9 male; placebo 5XFAD: 8 female and 6 male, pregabalin non-transgenic: 9 female and 8 male, pregabalin 5XFAD; 9 female and 10 male. At 21 weeks of age, mice were euthanized by ketamine/xylazine injection and transcardially perfused with PBS containing protease and phosphatase inhibitors. After perfusion, the brain was bisected, and one-half was dissected into the cortex, hippocampus, and cerebellum, and each region was separately flash-frozen in liquid nitrogen. The remaining half was fixed for 24 hours in 4% paraformaldehyde/PBS and cryopreserved in 30% w/v sucrose/PBS for sectioning. All animal work was carried out with the approval of the Northwestern University IACUC.

### 2.2. Quantification of Pregabalin in the Brain

Cerebellar tissue from pregabalin treated and untreated mice was shipped to Absorption Systems CRO (Exton, PA), along with a sample of pregabalin for the generation of the standard curve. Absorption Systems processed tissue and analyzed pregabalin content by LC-MS/MS methods.

### 2.3. Immunoblots

Cortices and hippocampi were processed in a NextAdvance Bullet Blender 24 Gold with glass beads in PBS/1% Triton X-100 containing protease inhibitors (Calbiochem Protease Inhibitor cocktail III) and Halt Phosphatase Inhibitor Cocktail (ThermoFisher Scientific) then were sonicated. Protein concentration was determined using the BCA assay (Pierce). 20 μg of homogenate was separated by 4-12% Nu-Page Bis-Tris gel in MES buffer (ThermoFisher Scientific). Protein was transferred onto 0.45 μm PVDF membrane, stained with 0.1% Ponceau, and then imaged. Blots were incubated with anti-APP antibody (6E10, BioLegend 803001, 1:2000), anti-BACE1 (3D5 [21], 1:1000), anti-LAMP1 (Cell Signaling Rabbit mAb #3243, 1:3000), anti-LC3B (Cell Signaling Rabbit mAb #3868 1:4000), anti-β-tubulin (TuJ1, gift of Dr. Nicholas Kanaan, 1:10,000) followed by HRP-conjugated anti-mouse or anti-rabbit secondary antibody (Vector Laboratories 1:10,000). 5% milk was used as a blocking agent. Blots were visualized using chemiluminescence (ECL+ or SuperSignal West Pico Plus, ThermoFisher), band intensities were measured using a FluorChemR imager (ProteinSimple), and then quantified with Alphaview software (ProteinSimple). Signal intensities were normalized to that of tubulin. Statistics were done as described below. For these analyses, multiple gels were cut into horizontal strips and stacked so that all samples for a given protein were transferred to a single piece of PVDF membrane. Putting all samples (up to 72) on one membrane eliminated the need to account for variation in transfer, antibody incubation, and ECL application that can occur between blots.

### 2.4. Dot Blotting

For Aβ42 dot blots, 5 mg/ml brain homogenates were extracted in 1.56 volumes of freshly made 8.2 M guanidine hydrochloride (GuHCl), 82 mM Tris HCl (pH 8.0) (5 M GuHCl final) over three nights on a nutator. 1 μl of GuHCl extracted sample (1.95 μg total protein) was spotted in triplicate on gridded nitrocellulose membrane, dried one hour at 37°C, washed in TBS-T and then water, and stained with Ponceau S. Four identical blots were made and incubated in either 1:4000 anti-Aβ42 rabbit monoclonal antibody (clone H31L21, Invitrogen, #700254), 1:4000 anti-Aβ total mouse monoclonal (3D6, Elan) or 5% milk only (primary delete), followed by HRP-conjugated secondary antibody (Vector Labs, PI-1000). The duplicate blots incubated with anti-Aβ antibody or primary delete were developed together with ECL Plus (Peirce) and imaged simultaneously using a FluorChemR imager (ProteinSimple), then quantified with Alphaview software (ProteinSimple). The secondary and primary antibody delete controls indicated no IgG background. Signal intensities were normalized to Ponceau S staining, triplicates were averaged, and statistics were performed as described below.

### 2.5. Immunostaining and Image Analysis

30 μm coronal floating brain sections were cut and placed in cryopreserve (30% w/v sucrose, 30% ethylene glycol in 1% PBS) in 12 well plates such that each well contained a series through the brain, with sections spaced 12 x 30 um (360 um) apart, then stored at −20°C. For staining, one well was moved to a cell strainer in a well of a six-well dish, rinsed three times in TBS, incubated in 16 mM glycine in Tris Buffered Saline with 0.25% Triton-X 100 (TBS-T), and then blocked firstly, with 5% donkey serum in TBS-T, and then with 1% BSA in TBS-T. Sections were incubated overnight at 4°C with 1:3000 rat monoclonal anti-LAMP1 (clone 1D4B, DSHB) and 1:3000 rabbit monoclonal anti-Aβ42 antibody (clone H31L21, Invitrogen, #700254) in 1% BSA TBS-T. The following day, they were incubated with 1:3000 donkey anti-rat Alexa 488 (ThermoFisher Scientific), donkey anti-rabbit 647 (ThermoFisher Scientific), 300 nM DAPI, and 1:30,000 dilution of 1 mg/ml Thiazine Red. All staining was performed at the same time. Sections were mounted with Prolong Gold (Molecular Probes) and images were acquired on a Nikon Ti2 Eclipse widefield microscope with a 10x objective, using NIS Elements software high content method to capture and tile whole sections. All image acquisition settings were the same for both the treatment groups and genotypes.

Three male and three female mice from each treatment group were selected for staining and image analysis. They were selected by choosing a mouse representing high, medi-um, and low BACE1 levels in the hippocampus by immunoblot, with the goal of correlating the extent of amyloid pathology with the varying levels of BACE1. Image analysis was performed in NIS Elements, using General Analysis. By using sections between −1.22 and −2.7 bregma, regions of interest (ROIs) were drawn in the cortex and hippocampus. By using the General Analysis tool, thresholding was set to distinguish Aβ42, Thiazine Red, and LAMP1 positive regions, then the percent area covered by each stain from the hippocampal or cortical ROI was calculated. By using the same sections, thresholding in the General Analysis tool was used to define sub-ROIs having both Aβ42 and LAMP1 positive pixels within cortical and hippocampal ROIs. These sub-ROIs correspond to individual plaques. The General Analysis tool was used to measure the area covered by Aβ42 and by LAMP1 in a given ROI (plaque), and the ratio between LAMP1: Aβ42 was calculated in Excel; 166-537 plaques were analyzed per mouse in the cortex and 95-225 in the hippocampus.

### 2.6. Statistics

Student's two-tailed t-test and ANOVA were done using InStat software (GraphPad Software, Inc., San Diego, CA) to compare means of the various genotypes, genders, and treatment groups. * 0.05 > p > 0.01 ** 0.01 > p > 0.001 *** 0.001 > p > 0.0001; Error bars = S.E.M.

## 3. RESULTS

### 3.1. Pregabalin is Well Tolerated and Detectable in Brain Tissue After 12 Weeks of Treatment

For investigating the effects of pregabalin on amyloid pathology, dystrophic neurite formation, and BACE1 elevation in AD, we used the 5XFAD transgenic mouse model of amyloidosis [43]. 5XFAD mice carry five mutations associated with autosomal dominant early-onset AD in humans. The transgene contains human APP with the K670N/M671L substitution (Swedish mutation [46]), the I716V substitution (Florida mutation [47]) and V171I substitution (London mutation [48]), and human PS1 with FAD mutations M146L and L286V, cointegrated under the control of the neuron-specific Thy-1 promoter. These mice have a very early onset of pathology, with plaques occurring at two months and increasing quickly with age. They also exhibit neuroinflammation, and neuronal loss [49].

**Fig. (1).**
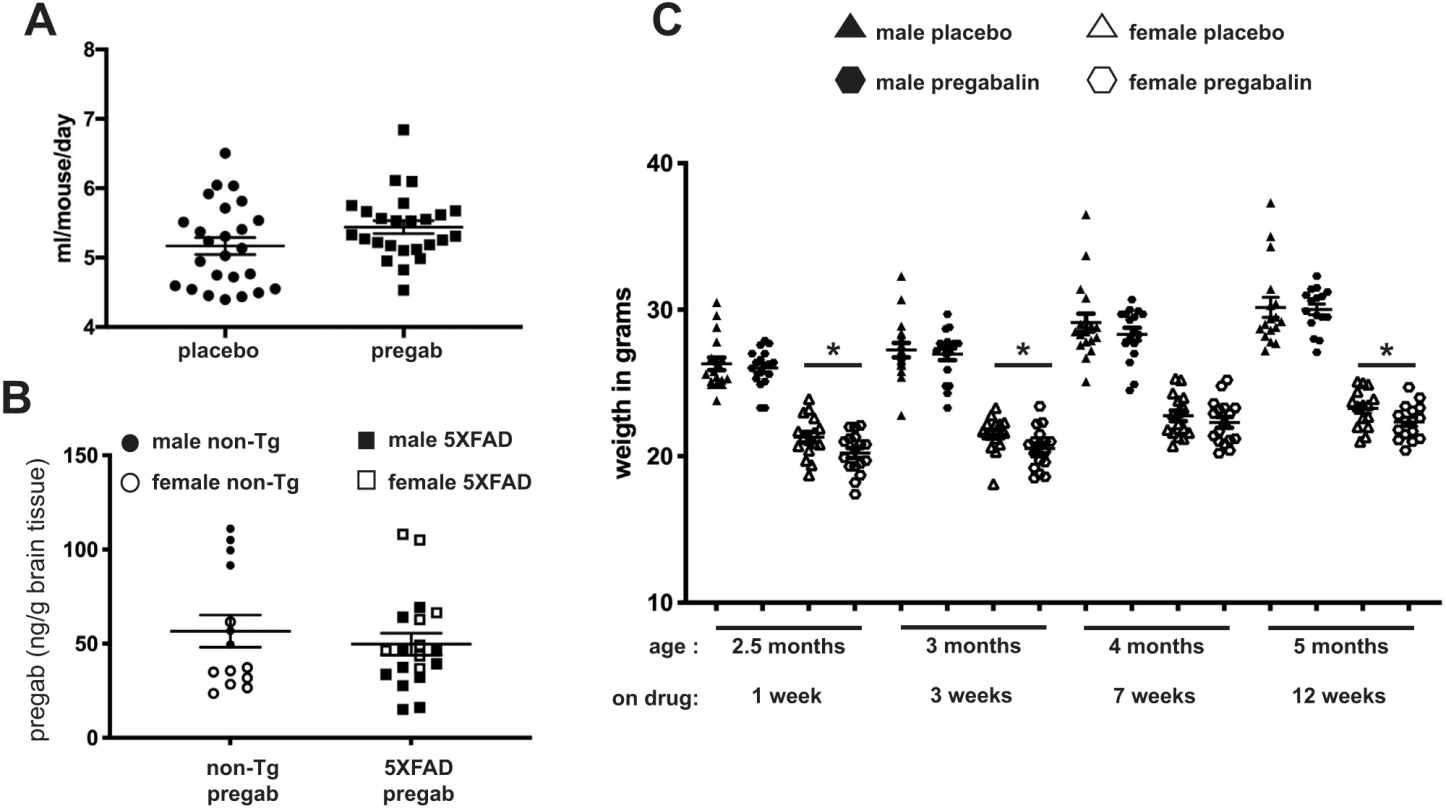
Pregabalin is well tolerated and penetrates into the brain. (**A**) Mice were provided with placebo and pregabalin containing drinking water from the ages of 9 weeks to 5 months. Over this time, mice receiving pregabalin and placebo consumed equivalent amounts of water per day. (**B**) Pregabalin was readily detected in cerebellar brain tissue and presented equal amounts in non-transgenic and 5XFAD mice. There was a notable animal to animal variation in concentration, with non-transgenic females having lower concentrations on average than non-transgenic males and 5XFAD males having lower concentrations than 5XFAD females on average. The amount of pregabalin was measured by mass spectrometry by Absorption Systems. (**C**) Over the course of the experiment, male mice that were administered pregabalin weighed the same as those consuming placebo. In females, mice that were administered pregabalin weighed less than those consuming placebo, except when they were 4 months old.

After the 5XFAD and non-transgenic littermate mice were 9 weeks old, they were treated with either 50 mg/kg/day pregabalin (the NOAEL for pregabalin in rats; [45]) or placebo in drinking water. The placebo used was the *R*-enantiomer of pregabalin ((*R*)-3-aminomethyl-5-methylhexanoic acid), which has a ten-fold decreased affinity for the α2-δ subunit, and no anti-epileptic, anti-anxiolytic, or anti-hyperalgesic effects *in vivo* models [44]. Although no sucrose or other flavor masking additives were used, mice drank equivalent amounts of pregabalin and placebo-treated water (Fig. 1A), within the normal range of water consumption for mice [50].

Pregabalin is known to cross the blood-brain barrier [51]. For determineing the concentration of pregabalin in the brains of treated mice, mass spectrometry was used to quantify drugs in cerebellar samples. A standard curve was generated using free compound, which was added to the cerebellum homogenate of a mouse that drank untreated water. Samples from seven mice given untreated water were all below the threshold of detection and lower than all samples from pregabalin-treated mice. There was no significant difference in pregabalin concentration between non-transgenic and 5XFAD mice or between males and females (Fig. 1B).

Mice were weighed periodically and assessed for any signs of drug toxicity, but all appeared healthy. The females on pregabalin water were significantly lighter than those on placebo water aged 2.5, 3 and 5 months, but not at the age of 4 months (Fig. 1C). Pregabalin and placebo-treated males showed no significant difference in weight at any time point, though there was a trend at all time points for the pregabalin-treated mice to be lighter.

### 3.2. Pregabalin Treatment does not Affect Biochemical Measures of Neuritic Dystrophy or Plaque Deposition

**Fig. (2).**
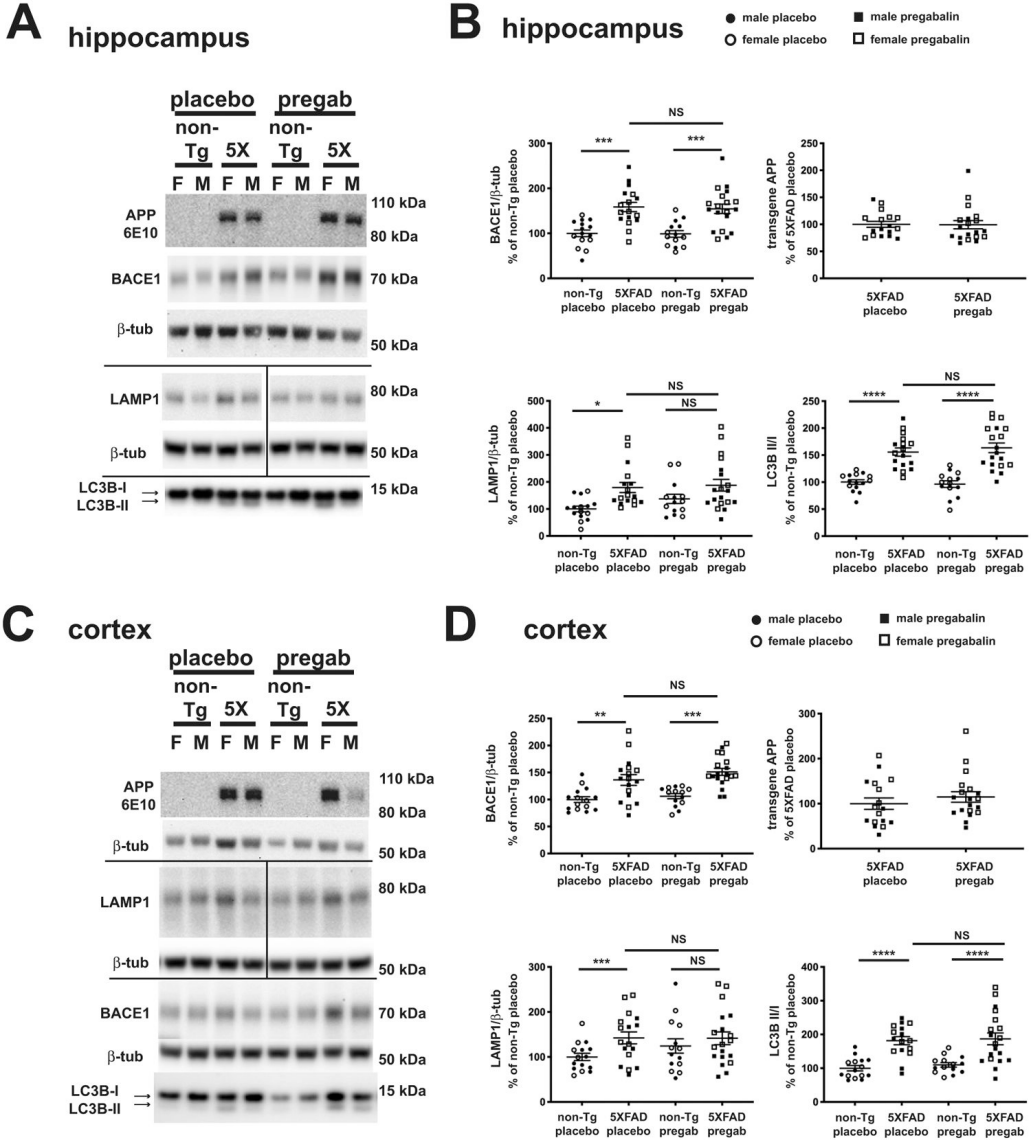
Levels of BACE1 and transgenic APP are not altered by pregabalin treatment. Sample immunoblots of BACE1, LAMP1, LC3B, and transgene-expressed human APP from hippocampal (**A, B**) and cortical (**C, D**) lysates; (**B, D**) quantification of immunoblot signals for BACE1, LAMP1, LC3B-I, LC3B-II, and APP; APP, BACE1, and LAMP1 are normalized to β-tubulin, while the LC3B-II:LC3B-I ratio is shown. As expected, BACE1, LAMP1, and LC3B-II/LC3B-I are elevated in 5XFAD placebo-treated mice compared to non-transgenic placebo-treated mice. BACE1 and LAMP1 are significantly elevated in 5XFAD pregabalin-treated mice compared to non-transgenic pregabalin-treated mice, and there is no significant difference between placebo- and pregabalin-treated 5XFAD for BACE1, LAMP1, or LC3B-II/LC3B-I. As expected, APP derived from the 5XFAD transgene was unchanged by the treatment with pregabalin. Similar results were observed in both hippocampus and cortex. Note: * 0.05 > p > 0.01 ** 0.01 > p > 0.001 *** 0.001 > p > 0.0001; Error bars = S.E.M.

**Fig. (3).**
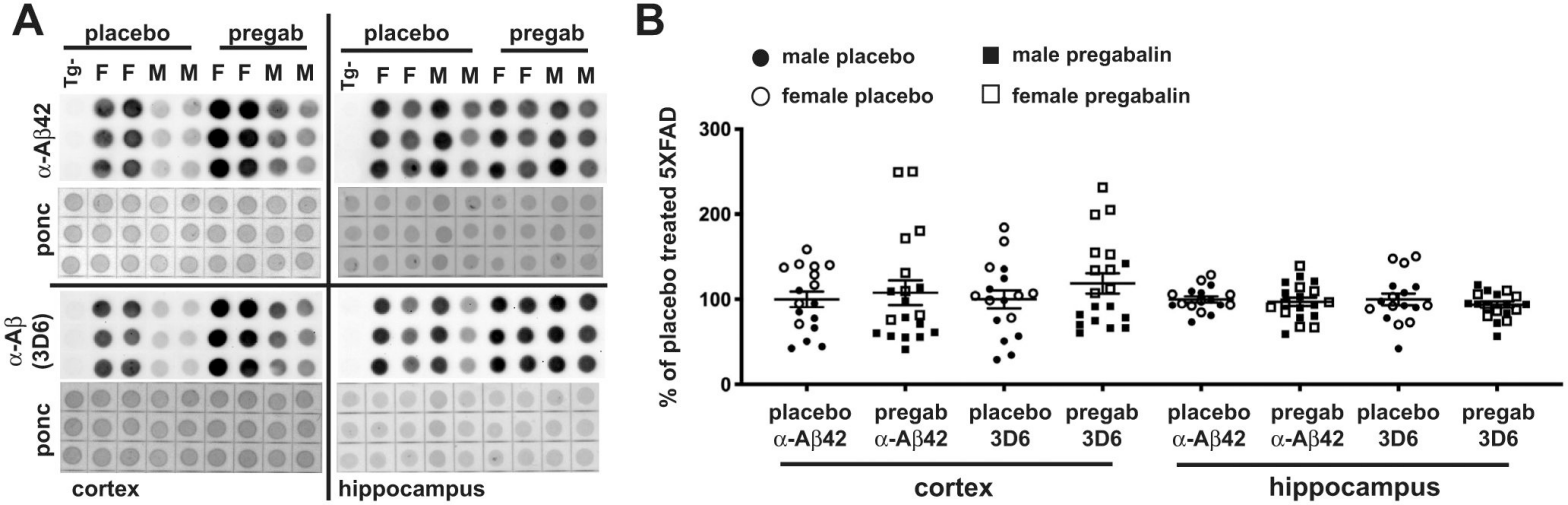
Aβ levels are not affected by pregabalin treatment. (**A**) Sample dot blots were analyzed using an Aβ42 specific antibody and a total Aβ antibody (3D6 from Elan) in the cortex and hippocampus. (**B**) The quantification of dot blots indicates no effect of pregabalin in either hippocampus or cortex on either Aβ42 or total Aβ species. There is a trend toward higher Aβ in females in the cortex. **Abbreviations:** Tg-: non-transgenic control, F: female; M: male, pregab: pregabalin treatment, ponc: ponceau staining.

At the age of 5 months, the mice underwent behavioral testing in Y-maze and fear conditioning, but no difference was observed between non-transgenic and 5XFAD mice treated with either placebo or pregabalin (Supplemental Fig. 1). This was unexpected, as previous work indicated deficits between five and six months of age in 5XFAD mice, but in the case of the fear conditioning, this may be related to differences in the timing of tone and shock in previous work [52]. After tissue harvest, cortical and hippocampal homogenates of pregabalin- and placebo-treated mice were examined by immunoblotting for the levels of BACE1, LAMP1, which accumulate in dystrophic neurites, LC3B, a marker of autophagy elevated in 5XFAD mice (Fig. 2), and the levels of Aβ were examined by dot blot (Fig. 3). In placebo-treated mice, BACE1 was elevated in 5XFAD compared to non-transgenic, which is in agreement with our previous reports [21, 53]. Another marker of dystrophic neurites, the early endosomal/lysosomal protein LAMP1 [54], was significantly elevated in placebo-treated 5XFAD compared to non-transgenic mice, as expected. In pregabalin-treated 5XFAD compared to non-transgenic mice, BACE1 was also elevated. We observed no difference in BACE1 elevation between placebo- and pregabalin-treated 5XFAD mice in either the hippocampus or cortex (Fig. 2B, D), indicating that pregabalin treatment did not reduce BACE1 accumulation in neuritic dystrophies. LAMP1 showed a non-significant trend for elevation in both placebo- compared to pregabalin treated non-transgenic mice and pregabalin treated 5XFAD compared to non-transgenic mice, though this did not reach significance because of the elevated LAMP1 in pregabalin treated non-transgenic mice. No difference in LAMP1 was observed between placebo- and pregabalin-treated 5XFAD mice in either the hippocampus or cortex (Fig. 2B, D). As expected, we observed a significantly elevated ratio of LC3B-II:LC3B-I, indicative of increased autophagy intermediates in 5XFAD mice compared to non-transgenic mice in both placebo and pregabalin treated groups [16]. We saw no difference between placebo- and pregabalin-treated 5XFAD mice in either the hippocampus or cortex (Fig. 2B, D), again suggesting that pregabalin has no effect on the accumulation of autophagic intermediates in dystrophic neurites. Brain homogenates were also assessed for the levels of transgenic APP using the antibody 6E10 that specifically recognizes human Aβ. There was no effect of pregabalin on transgenic APP, as expected, but as we have previously observed that females had higher levels of human APP [55]. This only reached significance (p = 0.012) in the cortex of the pregabalin-treated 5XFAD group. Together, these results suggest that pregabalin treatment does not reduce the biochemical markers of dystrophic neurites in 5XFAD mice, at least for the dose and treatment time used.

For assessing the levels of cerebral Aβ accumulation in placebo- and pregabalin-treated 5XFAD mice, guanidine hydrochloride treated brain homogenates were analyzed by dot blotting with two different anti-Aβ antibodies, one specific to Aβ42, and the other, 3D6, detecting total Aβ (Fig. 3). The two antibodies provided, as expected, very similar results. In the 5XFAD cortex, there was a higher mouse to mouse variation, with females having significantly higher amyloid load than males (3D6 cortex placebo: males *vs.* female (p = 0.02), pregab: males *vs.* females (p = 0.0001) and Aβ42 cortex placebo: males *vs.* female (p = 0.007), pregab: males vs. females (p = 0.018)). In the hippocampus, there was less variation, and male and female were not significantly different. In both hippocampus and cortex, there was no significant difference between Aβ levels of placebo-treated and pregabalin-treated 5XFAD mice, though there was a trend for increased Aβ in pregabalin-treated mice. When males and females were analyzed separately, there were still no significant differences, though there was a non-significant trend (p = 0.06) in the cortex for female pregabalin-treated mice to have higher Aβ measured by 3D6 than placebo-treated female mice. These results suggest that pregabalin treatment does not significantly affect cerebral Aβ accumulation in the brains of 5XFAD mice, at least for the dose and treatment time used.

### 3.3. Immunofluorescence Microscopy Confirms no Effect of Pregabalin Treatment on Amyloid Pathology

**Fig. (4).**
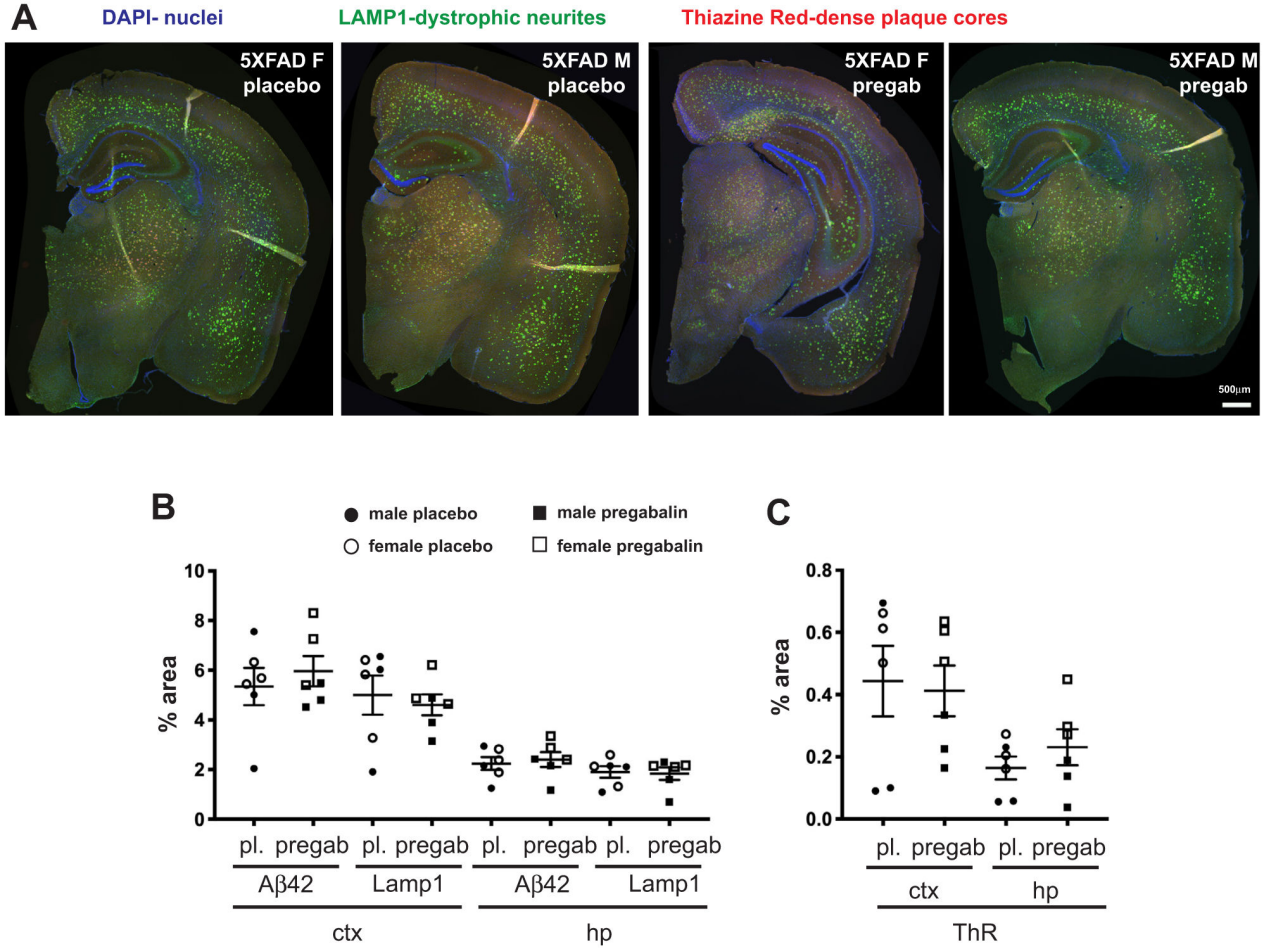
Percent area covered by Aβ42, LAMP1, and Thiazine red fluorescence signals are not affected by pregabalin treatment. (**A**) Representative coronal sections from the brains of 5-month-old 5XFAD positive mice stained with antibodies to LAMP1 to detect dystrophic neurites (green), Aβ42 to detect plaques (Fig. 5A), Thiazine Red to detect dense core plaques, and DAPI to stain nuclei. Sections were imaged at 20x on a Nikon Ti2 Eclipse microscope. Scale bar = 500 μm. (**B** and **C**). The total cortical or hippocampal area labeled for Aβ42, LAMP1 (**B**), or Thiazine red (**C**) was quantified for pregabalin and placebo-treated mice. Large animal-to-animal variation was observed, especially for amyloid pathology. No significant differences were observed between pregabalin and placebo- treated mice. **Abbreviation**: Pl: placebo, pregab: pregabalin, ctx: cortex, hp; hippocampus, ThR: thiazine red.

To determine whether pregabalin treatment had effects on BACE1 and neuritic dystrophy that might not be revealed by bulk biochemical analyses, we used immunofluorescence microscopy and the quantification of coronal brain sections from placebo and pregabalin-treated 5XFAD mice (Fig. 4). Three male and three female mice were selected from each treatment group for sectioning, staining, and image analysis for a more focused analysis. Using the immunoblot quantification of BACE1 in the hippocampus, we chose mice with the highest, lowest, and mean levels of BACE1 with the goal of determining whether differences in amyloid pathology were associated with given BACE1 levels. Floating sections (30 μm) were stained with antibodies against LAMP1, a lysosomal protein that accumulates in dystrophic neurites [54] and Aβ42 to visualize amyloid deposits. Sections were also stained with the fibrillar amyloid binding dye, Thiazine Red (ThR), to label dense core plaques. LAMP1 (green), ThR (red), and DAPI (blue) fluorescence signals in brain sections from 5XFAD mice treated with placebo or pregabalin revealed similar same-sex levels of neuritic dystrophy and amyloid pathology (Fig. 4A), with females tending to have greater amyloid burdens, as expected. When quantified, the percent areas occupied by Aβ42, LAMP1 (Fig. 4B), and ThR (Fig. 4C) were the same between placebo- and pregabalin-treated mice in both cortex and hippocampus. Plaque deposition covered a greater area in the cortex than the hippocampus, and there was more animal-to-animal variation in the cortex, with males lagging behind especially in dense core plaque deposition, as indicated by Thiazine Red positive area. The increased variability in Aβ levels in the cortex compared to the hippocampus was also observed by dot blot (Fig. 3B). Unexpectedly, the two males with the lowest levels of Aβ42, LAMP1, and ThR (one with placebo treatment, the other with pregabalin) did not have low BACE1, but rather high levels of BACE1 in the hippocampus by immunoblot analysis.

**Fig. (5).**
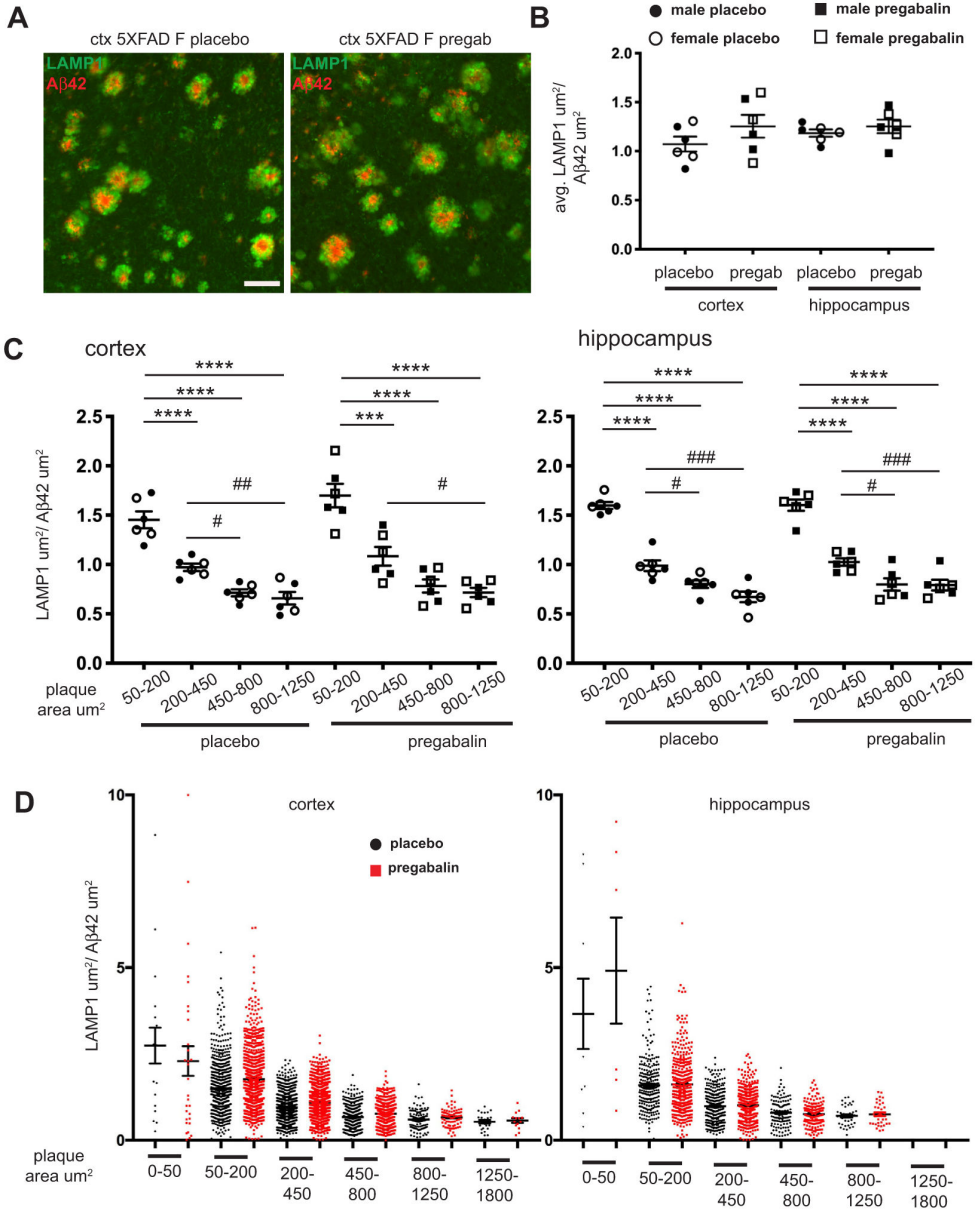
Neuritic dystrophy is not reduced in pregabalin-treated mice. (**A**) Representative images of cortical Aβ42 positive (red) plaques and LAMP1 positive (green) dystrophic neurite halos in placebo-treated (left) and pregabalin treated (right) mice. (**B**) Regions of the cortex and hippocampus were manually drawn on coronal sections imaged for Aβ42 and LAMP1 immunoreactivities (see Methods). Using Nikon NIS Elements software, individual plaques were defined as regions of interest (ROIs), and the areas positive for LAMP1 and Aβ42 within a given ROI were quantified in square microns. The ratio of LAMP1:Aβ42 was then calculated for each plaque and averaged from 166-537 plaques per mouse in the cortex and 95-225 plaques per mouse in the hippocampus. No significant difference in the average ratio of LAMP1:Aβ42 was observed in either the cortex or hippocampus of pregabalin- compared to placebo-treated mice. (**C**) Plaques were stratified by the indicated area ranges, and the average ratio of LAMP1:Aβ42 was calculated for each mouse within the area range. It should be noted that the average LAMP1:Aβ42 ratio is higher for smaller plaques in both cortex and hippocampus in placebo- and pregabalin-treated mice, indicating that small plaques cause the formation of dystrophic neurites more potently than large plaques. (**D**) LAMP1:Aβ42 area ratio for each plaque of a given area range was graphed for all mice that were treated with either pregabalin (red squares) or placebo (black circles). Plaques less than 50 μm^2^ were scarce, as were plaques 1250-1800 μm^2^, with the vast majority of plaques having an area between 50 and 800 μm^2^. Error bars = SEM. Scale bar = 50 μm.

For gaining more detailed information on the relationship between amyloid plaques and dystrophic neurites, and for testing our hypothesis that pregabalin would decrease neuritic dystrophy, we performed a further analysis in which regions of interest (ROI) in brain sections of placebo- and pregabalin-treated 5XFAD mice were defined as having both Aβ42 and LAMP1 positive pixels. This resulted in each Aβ42-positive plaque with LAMP1-positive dystrophic neurites being defined as a specific ROI. Example images of the LAMP1 and Aβ42 double-positive plaques/ROIs from the cortex are shown in Fig. (5A). Within each ROI, *i.e.*, plaque, the ratio of LAMP1 area to Aβ42 area was calculated. From a large number of plaques per animal, an average LAMP1:Aβ42 ratio was calculated for each mouse and graphed in Fig. (5B). We reasoned that the LAMP1: Aβ42 ratio should decrease if neuritic dystrophy was reduced on a per plaque basis. Conversely, an increased ratio would indicate that neuritic dystrophy was more extensive per plaque. Contrary to our hypothesis, we observed a non-significant trend for elevated LAMP1:Aβ42 ratio in the cortex of pregabalin treated mice compared to placebo (Fig. 5B).

Actively growing smaller plaques have been associated with proportionally larger dystrophic neurite halos compared to large plaques that exhibit slower growth [56]. For determining whether pregabalin could reduce neuritic dystrophy and if this was associated with plaque size, we analyzed the LAMP1:Aβ42 ratio in plaques stratified by size in cortex and hippocampus of pregabalin and placebo-treated mice (Fig. 5C). We found that the LAMP1:Aβ42 ratio was not significantly different between placebo and pregabalin groups for any plaque size, but we observed a non-significant trend for an increased ratio in pregabalin-treated brains for the smaller plaques of 50-200 μm^2^ and 200-450 μm^2^ in diameter in the cortex; no such trend was seen in hippocampus.

Interestingly, we observed that larger amyloid plaques have proportionally less LAMP1-positive neuritic dystrophy than smaller deposits in both cortex and hippocampus. In the cortex, no statistically significant difference was found for LAMP1:Aβ42 area ratio between placebo- or pregabalin-treated mice for the larger plaques (450-800 μm^2^ and 800-1250 μm^2^). However, plaques of 50-200 μm^2^ had a significantly higher ratio than all other plaque area ranges; 200-450 μm^2^ plaques had a ratio that was significantly greater than the ratio of 450-800 μm^2^ and 800-1250 μm^2^ plaques in the placebo group and 800-1250 μm^2^ plaques in the pre-gabalin treated group (cortex one way ANOVA placebo F (3, 20) = 37.23, p<0.0001, pregabalin F (3, 20) = 26.84, p<0.0001) (Fig. 5C). A similar pattern was observed in the hippocampus, with plaques of 50-200 μm^2^ having a significantly higher ratio than all other plaque area ranges, and 200-450 μm^2^ plaques having a ratio significantly greater than 450-800 μm^2^ and 800-1250 μm^2^ plaques in the placebo and pregabalin treated groups (hippocampus one way ANOVA placebo F (3, 20) =78.5, p<0.0001, pregabalin F (3, 20) = 50.38, p<0.0001) (Fig. 5C). These results are congruent with a previous report using a time-stamp methodology to show that small plaques have faster rates of growth and that increased growth rate is correlated with greater neuritic dystrophy 56. Another advantage of analyzing each plaque individually was that we obtained a size distribution of the plaques with neuritic dystrophy, as shown in Fig. (5D). As mentioned, plaques under 50 μm^2^ were infrequent, as were those between 1250-1800 μm^2^; in fact, this larger class was absent in the hippocampus. Since this only included mice aged 5 months, it is not clear whether hippocampal plaques are simply lagging behind cortical plaques in growth or if they will remain smaller even as pathology progresses.

### 3.4. Higher Levels of Pregabalin in the Brain Correlate with Higher Aβ Levels

**Fig. (6).**
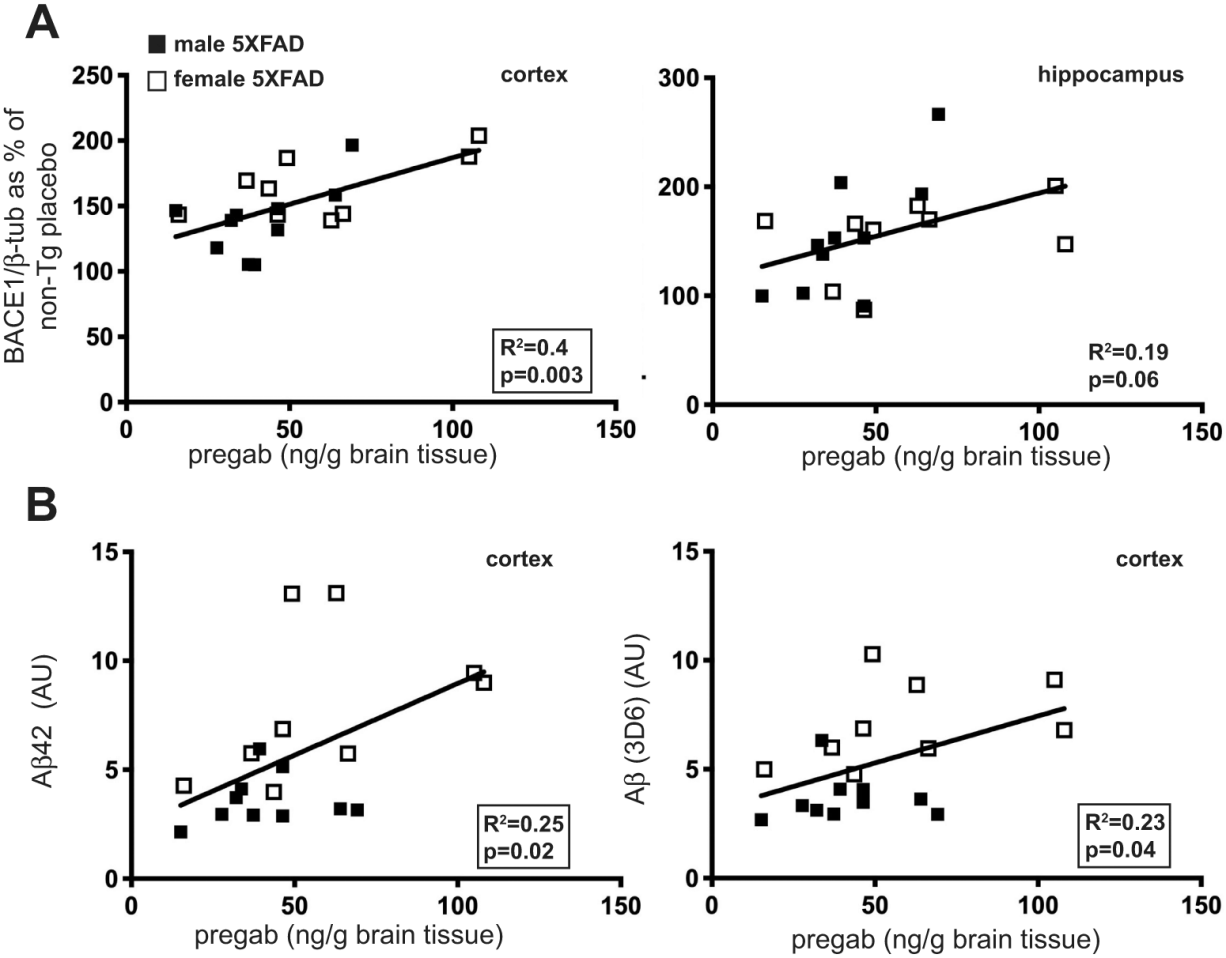
Cortical BACE1 and Aβ levels positively correlate with cerebral pregabalin concentrations in 5XFAD transgenic mice. (**A**) Linear regression analysis of BACE1 level (% of non-Tg placebo, as determined by immunoblot analysis, Fig. 2) and cerebral pregabalin concentration (ng/g brain tissue, as determined by mass spectrometry, Fig. 1B) for cortex (left panel) and hippocampus (right panel). A significant correlation was found between pregabalin concentration and BACE1 level in the cortex, but not in the hippocampus, of treated 5XFAD mice. (**B**) Linear regression analysis of Aβ (AU, as determined by dot blot, Fig. 3B) and cerebral pregabalin concentration (ng/g brain tissue, as determined by mass spectrometry; Fig. 1B) in the cortex for Aβ42 (left panel) and total Aβ (3D6 antibody) (right panel). A significant positive correlation was observed between Aβ level and pregabalin concentration in the cortex but not in the hippocampus (data not shown). **Abbreviation**: AU: arbitrary units, non-Tg: non-transgenic, pregab: pregabalin.

Since individual mice exhibited a wide range of pregabalin concentrations in the brain (~25-100 ng/g brain tissue; Fig. 1B), we determined whether cerebral BACE1 and pregabalin levels were correlated. to accomplish this, we performed a linear regression analysis of BACE1 levels in cortex and hippocampus and pregabalin brain concentration of pregabalin-treated 5XFAD and non-transgenic control mice (Fig. 6A). Surprisingly, we observed a positive correlation between BACE1 and pregabalin in the cortex but not in the hippocampus (p = 0.06) (Fig. 6A), suggesting that pregabalin treatment may elevate BACE1 in certain brain regions. This correlation was only observed in 5XFAD transgenic mice, not non-transgenic mice (data not shown). Since BACE1 is the enzyme responsible for initiating the cleavage cascade that leads to the generation of Aβ from APP, we performed a similar linear regression analysis of Aβ level in cortex and hippocampus and pregabalin brain concentration to determine if pregabalin concentration affected amyloid levels (Fig. 6B). In the cortex, there was a correlation between pregabalin and Aβ42 as well as total Aβ (Fig. 6B), but in the hippocampus, no correlation was observed (data not shown, pregabalin *vs.* Aβ42: R^2^ = 0.02, p = 0.6; pregabalin *vs.* Aβ, (3D6): R^2^ = 0.1, p = 0.17). Given this correlation between pregabalin concentration and Aβ, in the cortex, it was unexpected that the dot blot and immunoblot results did not show an elevation of Aβ or BACE1 in the cortex of pregabalin treated mice. However, it may be that the effects of the highly variable pregabalin levels in the brain could have masked an increase when the values from all the mice were averaged. These results suggest the possibility that long-term treatment with pregabalin could lead to increased Aβ, deposition and increased BACE1 accumulation. However, this possibility seems unlikely, as the recommended therapeutic dosage of pregabalin is in the range of 150-660 mg/day (2.2 mg/kg/day - 9.7 mg/kg/day assuming 150 lb l), which is much less than the 50 mg/kg/day dosage administered to mice in our study.

## 4. DISCUSSION

In this report, we have assessed the ability of a 12-week course of treatment with pregabalin to slow or delay the development of dystrophic neurites and amyloid plaques in the 5XFAD mouse model of AD amyloidosis. We show that while 50 mg/kg/day pregabalin administered in drinking water is well tolerated and results in pregabalin accumulation in brain tissue, we observed no reduction of BACE1 by immunoblot assay in brains of pregabalin-treated mice compared to controls. In addition, we used both biochemical assays and immunofluorescent imaging methods to measure amyloid pathology and neuritic dystrophy and found no significant changes caused by pregabalin treatment using either method; only a trend for increased LAMP1-positive dystrophies around smaller plaques was observed. Surprisingly, we observed that the concentration of pregabalin measured in the brain correlated positively with the level of BACE1 and Aβ in the cortex but not the hippocampus. This is disappointing from the point of view of using pregabalin as an alternative AD therapeutic to target BACE1 elevation and dystrophic axons and suggests further study of the use of pregabalin in AD patients to assess effects on pathology. Two of the indications for pregabalin use, neuropathic pain due to diabetes, and epilepsy are conditions more prevalent in AD patients than in the general population. Diabetes is a risk factor for developing AD [57], as is epilepsy [58]; the rates of epilepsy in AD patients are higher than matched controls [59]. However, a recent large study using data from the Finnish Patient register and German health insurance databases indicated no increased risk of dementia or AD based on previous pregabalin use [58].

Our work also demonstrates ways in which detailed image analysis can provide insight into subtle pathologic changes. Stratifying plaques by area provided additional information about how the ratio of LAMP1-positive to Aβ42-positive areas varies with plaque size and with brain region. In future studies, it will be useful to use this technique to determine how the average ratio changes for different plaque sizes over the course of pathological progression. It may be that the LAMP1:Aβ42 ratio for a given plaque area remains constant, but the relative distribution of plaque areas may change with age, with a greater representation of larger plaque areas in later disease stages. In the current analysis, plaques under 50 μm^2^ were not analyzed because their low numbers were not sufficient for statistical analysis. It was somewhat surprising that plaques under 50 μm^2^ were scarce since new plaques should still be forming in 5XFAD mice at 5 months of age. One possible explanation for the low number of small (< 50 μm^2^) plaques in our study may be that the smallest plaques have not yet formed many dystrophic neurites, as these structures follow the appearance of amyloid deposits [31] and thus may be missed when only analyzing objects containing both Aβ42 and LAMP1. Longitudinal live imaging found that the average area of a new plaque detected using methoxy-XO4 was 50 μm^2^ in the APPswe/PS1d9 mouse model of amyloidosis, indicating that this size range likely represents the youngest plaques [31]. A later study, using Vesicular Glutamate Transporter 1 (VGLUT1) as a marker for dystrophic neurites, found that plaques with a radius smaller than 4 μm (equivalent to an area of 50 μm^2^) did not tend to have dystrophic neurites in APPPS1-21 mice [24], which may account for the low number of plaques with an area less than 50 μm^2^ that were associated with LAMP1 positivity in our study. However, although we observed a high level of variability in the LAMP1:Aβ42 ratio for 0-50 μm^2^ plaques, they have on average the greatest LAMP1 in proportion to Aβ42, suggesting new plaques are proportionally more potent at inducing dystrophic neurites. Finally, we cannot eliminate the possibility that the centers of some plaques were not contained within the brain section, thus causing skewing of LAMP1:Aβ42 ratios toward artifactually high values. Although this scenario is unlikely to affect our current results or conclusions in major ways, in future studies, we will analyze high-resolution z-stacks of plaque images in order to assess three-dimensional plaque structures and ensure that LAMP1:Aβ42 area ratios are measured for plaques at their maximum diameters.

There are several possible reasons that the pregabalin treatment had no detectable effect on dystrophic neurites. When pregabalin was measured in the brain, the average concentration of 50 ng/g in brain tissue was twenty-fold lower than the 100 μg/g associated with complete seizure prevention in rats after a single 5 mg/kg pregabalin intravenous injection [60]. The possibility exists that higher pregabalin brain concentrations are required to reduce resting intraneuronal calcium levels in presynaptic compartments sufficiently to decrease dystrophic neurite formation. Alternatively, the binding of pregabalin to the α2-δ subunit of presynaptic P-type calcium channels inhibits calcium influx in response action potentials, leading to reduced neurotransmitter release and less downstream excitability without affecting resting calcium levels, thus having no effect on microtubules or vesicle trafficking. Further work to measure calcium levels *in vivo* in brains of mice treated with pregabalin compared to placebo will help determine whether resting calcium is decreased in cells and dystrophic neurites, or if principally the firing patterns of cells are changed. These experiments are technically challenging as calcium sensors are usually designed to measure the change in calcium concentration over time rather than compare calcium concentrations in different regions. Normalizing differential accumulation of calcium-sensitive dyes or proteins can be challenging *in vivo* as well. Immunoblotting for calcium-sensitive phospho-epitopes in placebo and pregabalin-treated brains did not indicate decreased calcium levels in pregabalin-treated brains, but the bulk approach of immunoblotting may miss more subtle localized changes. Since we did not test for known effects of pregabalin, such as seizure prevention, we cannot be certain that the levels achieved in this experiment were high enough for biological activity.

Other anti-epileptics have been demonstrated to decrease Alzheimer’s related pathology in mouse models. Valproate has been reported to decrease amyloid plaque deposition and tau phosphorylation, ameliorate cognitive deficits, and increase neurogenesis in various mouse AD models [61-63]. Topiramate has similar effects [64] as do carbamazepine [65] and levetiracetam [66], but all these effects may be caused by the enhancement of autophagy and/or the inhibition of histone deacetylase, not by the drugs’ main mechanism of calcium channel or sodium channel modulation [65, 66]. Since many anti-epileptics have multiple targets and a wide range of mechanisms of action [67, 68], it is difficult to determine which pathways are exerting the beneficial effects on AD pathology.

Although pregabalin contains the GABA backbone (γ-aminobutyric acid), it does not bind to GABA receptors or transporters [69] and appears to have no effect on the level of GABA in the brain, though it can decrease glutamate levels [70], possibly by affecting glutamate transporter activity [71]. Generally, decrease in the synaptic activity reduces Aβ production [72], therefore, it is surprising that higher levels of pregabalin were correlated with elevated Aβ levels in the cortex (Fig. 6B). However, it is important to point out that no statistically significant increase in histological or Biochemicalcal measures of Aβ were observed in pregabalin-treated 5XFAD mice. In the future, the analysis of cell culture and mouse models will be valuable to address the molecular mechanisms by which pregabalin could potentially increase Aβ, possibly by affecting degradation or clearance and might also provide insight into novel pathways of Aβ regulation that may have therapeutic implications. The study of human-derived cells is also be necessary as the effects of pregabalin on BACE1 levels in mice may not fully predict what happens in humans since aspects of the regulation of BACE1 expression may vary in the two species.

Dystrophic neurites are directly caused by amyloid plaques, as clearance of Aβ deposits *via* immunotherapy results in the reversal of plaque-associated dystrophies [32]. Additionally, the compaction and shielding of plaques by microglia appeared to protect nearby axons and dendrites and prevent neuritic dystrophy [73, 74]. In mice with either the deletion of or the introduction of AD-associated mutations in the AD risk gene TREM2, microglia failed to migrate to and wall-off plaques, which were less compacted and had a higher degree of neuritic dystrophy [73, 74]. Conversely, increased expression of TREM2 in 5XFAD mice led to a reduction in dystrophic neurite volume in the brain [75]. In this study, amyloid was also decreased, therefore, there may be a possibility that the neuritic dystrophy reduction just reflected the decreased amyloid, not a reduction in the amount of dystrophy per plaque, though the decrease in neuritic dystrophy (50%) was greater than the reduction in amyloid (25%) [75]. Recent work suggests that changes to the cytoskeletal structure in the dystrophic neurites may be able to reduce BACE1-positive dystrophies and Aβ generation. APPPS1-21 mice lacking Tau show reduced BACE1 accumulation in dystrophic neurites and reduced plaque seeding [76].

## CONCLUSION

Given these observations, it may be necessary to address these molecular and cellular pathways in addition to the reduction of intracellular calcium levels to have an effect on dystrophic neurite formation.

## Supplementary Material

Sadleir_PMID34259145_CurrAlzRes_2021
