# Supplementary material for: Pregabalin Treatment does not Affect Amyloid Pathology in 5XFAD Mice
Source: Curr Alzheimer Res. Author manuscript; Available in PMC 2022 Oct 3. (PMC9527523; doi:10.2174/1567205018666210713125333)
Supplement: Sadleir_PMID34259145_CurrAlzRes_2021 [file NIHMS1837739-supplement-Sadleir_PMID34259145_CurrAlzRes_2021.pdf]

Supplementary Material

Pregabalin Treatment does not Affect Amyloid Pathology in 5XFAD Mice

Katherine R. Sadleir[1,*], Jelena Popovoic[1], Wei Zhu[2], Cory T. Reidel[2], Ha Do[2], Richard B. Silverman[2,3] and Robert Vassar[1]

[1]Dept of Neurology, Feinberg School of Medicine, Northwestern University, Chicago, IL, USA; [2]Dept of Chemistry, Chemistry of Life Processes Institute, Center for Molecular Innovation and Drug Discovery, Center for Developmental Therapeutics, Northwestern University, Evanston, IL, USA; [3]Dept of Pharmacology, Feinberg School of Medicine, Northwestern University, Chicago, IL, USA

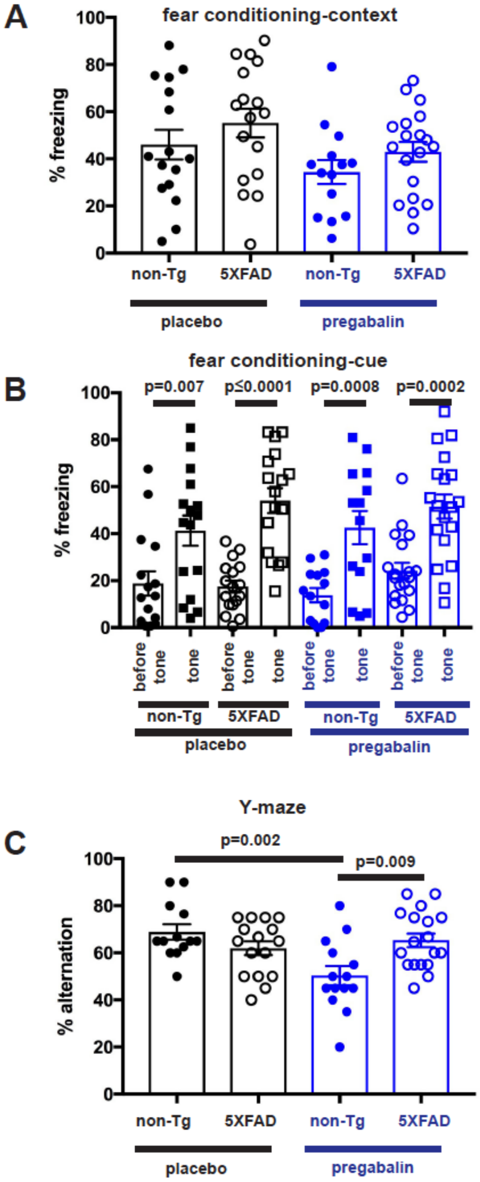

Supplemental Fig. (1). Five month old 5XFAD mice do not show behavioral deficits. A) When placed in the same context after training, all groups (non-Tg and 5XFAD treated with either vehicle or placebo) show equal amounts of freezing behavior, with high interindividual variability. **B**) When placed in a novel chamber after training, initial rates of freezing were low for all groups (15-25%) with no significant difference between groups. During the conditioned tone, all groups significantly increased freezing behavior compared to time before tone, though there was no significant difference in percent freezing between groups. **C**) In the Y maze, the non-Tg pregabalin treated groups had significantly lower precent alteration than non-Tg placebo and 5XFAD prebagalin, though we do not know why.
